# Systematic review: differences in complete blood count component rhythms

**DOI:** 10.1093/sleepadvances/zpae086

**Published:** 2024-12-09

**Authors:** Anna Busza, Vani Sharma, Kendall Ferguson, Andrea Fawcett, Justin Knoll, Marta Iwanaszko, Phyllis Zee, Anna Fishbein

**Affiliations:** Department of Pediatrics, Northwestern University Feinberg School of Medicine, Chicago, IL, USA; School of Molecular and Cellular Biology, College of Liberal Arts and Sciences, University of Illinois at Urbana Champaign, Champaign, IL, USA; Department of Biology, Northwestern University, Evanston, IL, USA; Department of Clinical and Organizational Development, Ann & Robert H. Lurie Children’s Hospital, Chicago, IL, USA; Department of Medical Social Sciences, Northwestern University, Feinberg School of Medicine, Chicago, IL, USA; Department of Biochemistry and Molecular Genetics, Northwestern University Feinberg School of Medicine, Chicago, IL, USA; Department of Neurology and Center for Circadian and Sleep Medicine, Northwestern University Feinberg School of Medicine, Chicago, IL, USA; Department of Allergy, Division of Pediatric Allergy and Immunology, Ann & Robert H. Lurie Children’s Hospital, Chicago, IL, USA

**Keywords:** circadian rhythm, biological rhythms, statistics, diurnal rhythm, complete blood count, reference values

## Abstract

**Study Objectives:**

The complete blood count (CBC) is one of the most commonly ordered blood tests with a large range of reference values that does not consider time of day for interpretation. Our objective was to systematically review this topic to report on peak and trough timing of CBC values.

**Methods:**

A systematic search was performed for studies evaluating any component of the CBC with at least three collections over 24 hours. The studies were screened based on the predetermined eligibility criteria. Meta-analysis of aggregated data was analyzed with polynomial functions and forest plots.

**Results:**

In total, 164 full-text articles were screened and 32 included in the final analysis with 548 total patients considering either leukocytes (*n* = 13), erythrocytes (*n* = 7), hemoglobin (*n* = 5), hematocrit (*n* = 5), platelets (*n* = 12), neutrophils (*n* = 11), lymphocytes (*n* = 13), monocytes (*n* = 8), eosinophils (*n* = 15), or basophils (*n* = 9). CBC components were analyzed by polynomial and forest plot analysis. Lymphocytes fitted best to a third-degree polynomial function (*p* = .010) with peak at 2264.87 cells/µL at 23:54 (CI: 1783.44 to 2746.31) with a trough of 1598.91 cells/µL at 10:47 (CI: 1230.12 to 1967.71). Lymphocytes and eosinophils peaked overnight, while erythrocytes, hemoglobin, and hematocrit peaked in the morning, and platelets, neutrophils, monocytes, and basophils peaked in late afternoon. Limitations include small sample size and significant study heterogeneity.

**Conclusion:**

We identified a limited scope of studies characterizing CBC component rhythms. However, we still noted significant differences, particularly with lymphocytes. Future work should evaluate larger datasets to inform time-dependent interpretation of the CBC as we move toward precision medicine.

**Statement of Significance**

The complete blood count (CBC) is one of the most commonly ordered blood tests with multiple cell components having time-dependent differences of potential clinical significance. Most physicians are unaware of these changes to the components of the blood count and these fluctuations may have clinical significance. Although many individual studies have evaluated time of day differences, to our knowledge, our study is the first systematic review/meta-analysis on this topic. With 32 available studies and 548 total patients, we quantified maximum and minimum levels and timing of each cell type in the CBC. Future work with larger data sets is needed to develop reference values reflective of time-dependent behavior of the CBC.

## Introduction

Circadian rhythms are the endogenous 24-hour oscillatory behaviors in physiology that allow humans, and other organisms, to anticipate changes in their environment and react appropriately [1]. The most commonly known example is the sleep–wake cycle, but circadian patterns exist in many other aspects of our biology including our body temperature, hormone release, and blood counts [1]. When these 24-hour behaviors are controlled by both endogenous and exogenous (e.g. environmental) cues, this is termed a diurnal rhythm [1]. Circadian rhythms are a subset of diurnal rhythms defined as rhythms that are set endogenously and persist without synchronization to environmental cues [1]. Although the diurnal rhythm of blood cells has been known since the last century with numerous investigations aiming to elucidate the diurnal behavior of the hematologic system, this factor is not accounted for clinically [2]. Specifically, there continues to be a gap of knowledge with regards to the expected timing of peak/trough values and the degree to which values might fluctuate and greater precision could thereby improve medical diagnostics.

The complete blood count (CBC) is one of the most commonly ordered laboratory tests clinically and measures the cell count of erythrocytes, leukocytes, hemoglobin, hematocrit, neutrophils, lymphocytes, eosinophils, monocytes, and basophils per microliter of blood. Research to establish accurate CBC reference values for different populations, ages, and devices continues to be ongoing, yet these studies almost always overlook the effect of the circadian system on blood cells reference values [3–5].

With regards to previous work on the topic, there are several review articles which consider circadian mechanisms underpinning the hematologic system [6–8]. A previous scoping review on leukocyte counts provides changes over 24 hours presumably from a central laboratory repository of samples [9]. However, this study did not consider blood draws from the same participants at multiple times [9]. A previous retrospective review on leukocyte subsets analyzed interpatient differences of diurnal blood count values but was limited to day time blood draws and also did not compare the results of blood draws from the same patient [10]. To our knowledge, there are no systematic reviews/meta-analyses considering studies of the CBC in which multiple blood draws occurred from the same subject over time.

There has been recent discourse by Burack and Lichtman (2023) discussing the redundancy of certain components of the CBC, and responses reinforcing the utility of the CBC for patient referrals and subsequent diagnoses [11]. In the age of precision medicine, technology is available to further specify the reference intervals the CBC to allow clinical implementation in improving diagnostic capacity [8, 12]. This gap in knowledge can be filled.

In this systematic review, our objective was to identify the scope of previously published studies on the diurnal rhythm of specific components of the CBC. The goal of our meta-analyses of these specific components was to provide clinicians with preliminary tangible parameters by which to consider time of collection in interpretation of the CBC.

## Methods

The systematic review followed the guidelines and statement criteria established by Preferred Reporting Items for Systematic Reviews and Meta-analysis [13]. We searched PubMed, Ovid, Scopus, CINAHL, Cochrane, Embase, and Web of Science databases from inception to June 5, 2022. Database searches took place the week of June 5, 2022. Search terms included “Circadian Rhythm,” “acrophase,” “time of day,” and “CBC.”

Search strategy developed by our study team (including reference librarian Ms. Andrea Fawcett) was published in PROSPERO and can be accessed at https://www.crd.york.ac.uk/prospero/display_record.php?RecordID=308435. Ms. Andrea Fawcett conducted the database searches and de-duplicated articles. The abstracts were then reviewed by two authors independently using Rayyan software for the following criteria. Briefly, included studies contained primary or secondary outcomes either including a comparison of three or more values from a CBC over 24 hours in healthy adults (18–64 years of age) or adults with asthma or the development of circadian rhythmicity of CBC values. Abstracts and studies which included only specific subsets of a cell type component in a CBC (such as T-cell counts but not lymphocyte counts) were excluded.

The process of quality appraisal, data extraction, and analysis was guided by the National Institutes of Health (NIH) Quality Assessment Tool for Observational Cohort and Cross-Sectional Studies and the NIH Quality Assessment of Case-Control Studies [14]. Two authors independently reviewed all eligible articles in order to select only those fulfilling the inclusion criteria. Any disagreement during this process was resolved by consultation with a third reviewer. Any article written in a language other than English was translated by an additional reviewer fluent in that language. With the final set of articles, two authors extracted the relevant data into a preformed extraction data document. If the article was unclear or the data for our primary outcome was not included, contact with the author was attempted.

To answer the primary research question, primary outcomes for this study were CBC values of each cell type with at least three time points reported over 24 hours or circadian behavior of CBC cell types described over 24 hours. Three potential data reporting methods were recorded: (1) Peak and trough times of CBC cell types over 24 hours, (2) Data points over 24 hours for cohort or individual participants, and (3) Circadian characteristics of CBC cell types over 24 hours. Some studies included two or more of the data reporting methods and both were included in the data extraction and analysis. Details on the study type, location, and experiment design were recorded. Controlled circadian factors (lighting, inpatient or outpatient setting, activity level, meal times, blood draw conditions, and sleep times) were also recorded in detail as well as participant characteristics (age, sex, and health).

To aggregate results across studies, initially, descriptive statistics were used. Within each study, peak and trough values were averaged across all included participants at each time point. There were no instances of multiple time-point values for the same participant. Each study provided data either with a cosinor function and/or with an output of the composite peak and trough values and times. The peak and trough values and times of the cosinor function were then utilized to compare values across studies. Next, a meta-analysis via forest plot analysis was conducted with average peak and trough values of each component of the CBC from studies that included standard error or standard deviation (Stata SE) [15]. For each component of the CBC, average timing of peak/trough values were computed using a weighted average via circular statistics (Excel). Finally, a first-, second-, or third-degree polynomial function was fitted for each CBC cell type to assess rhythmicity. Analyses were conducted using “broom” and base packages, then visualized using the ggplot2 with linear model (lm) smoothing in the R statistical environment [16]. We systematically compared the goodness of fit for the first-, second-, and third-degree polynomial models by utilizing both the statistical significance of model coefficients as well as additional goodness-of-fit metrics such as *r*^2^, adjusted *r*^2^, AIC, and BIC. For each dataset, we first performed likelihood-ratio tests to determine whether increasing the polynomial degree significantly improved the model fit. Only if the higher-degree polynomial demonstrated better *r*^2^ and a better statistical significance it was chosen over a simpler model.

## Results

### Study selection

**Figure 1.**
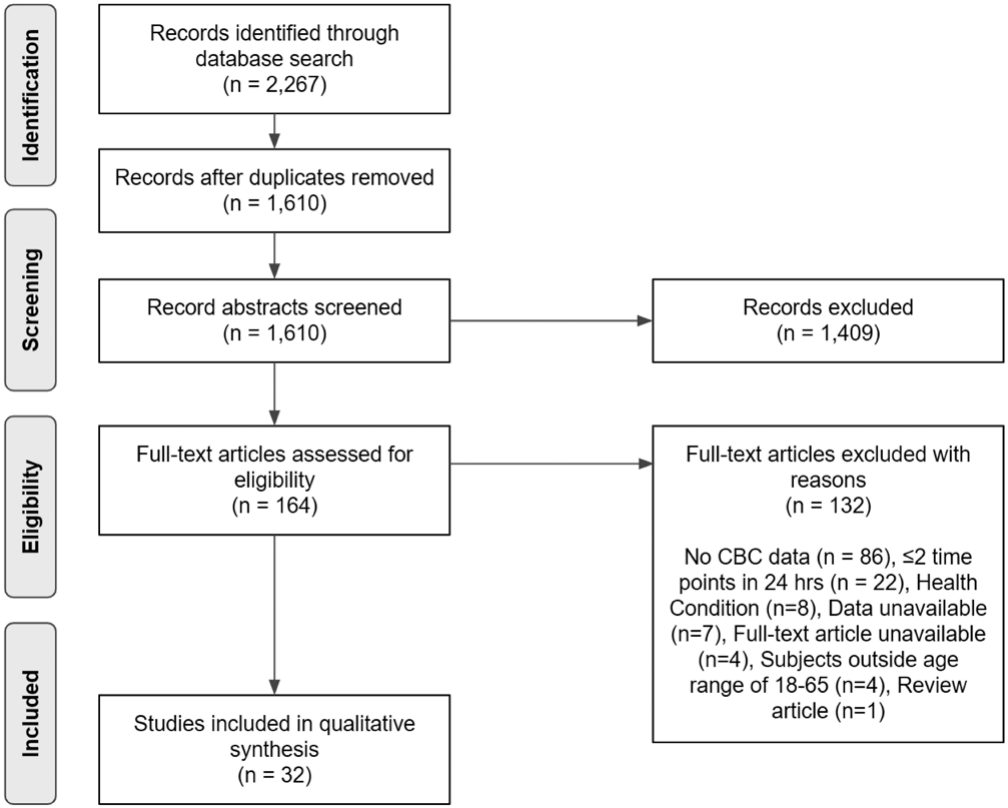
Preferred Reporting Items for Systematic Reviews and Meta-Analyses flow diagram. This diagram depicts the search strategy and methodology for selecting articles that were included in the final analyses. The diagram shows the total number of records identified through database searching (*n* = 2,267). After removing duplicates, the number of records screened (*n* = 1,610) is shown, with the number of records excluded at this stage (*n* = 1,409). The diagram then presents the number of full-text articles assessed for eligibility (*n* = 164), and the number of studies excluded with reasons (*n* = 132). Finally, the diagram indicates the total number of studies included in the qualitative synthesis (*n* = 32). The four unavailable full-text articles included one Bulgarian, one German, one Russian, and one English publication [17–20].

Study selection is outlined in Figure 1. Briefly, the initial electronic database search through May 6, 2022 resulted in 2267 hits. In addition, one record was found in the reference list of an identified study. After careful examination, 1409 records were excluded for not meeting the inclusion criteria based on the title and abstract, with 164 articles selected for full-text screening.

Thereafter, 132 articles were removed for the following reasons: 86 did not include CBC data; 22 had two data points or fewer over 24 hours; eight included patients with health conditions (exception: asthma for papers regarding eosinophils); seven required the full data to determine the eligibility and the authors did not respond to email; four full-text articles were unavailable; four had patients outside the defined age range of 18–65 years old; and one was a review article. In the end, 32 articles were identified that met our a priori published inclusion criteria, as well as the additional criteria of including data for three or more time points.

### Study description

Table 1 describes the 32 studies included for detailed review. The number of patients per study varied from 2 to 150. Years of publications ranged from 1969 to 2021. Studies were all prospective observational cohorts.

**Table 1.** Studies included in the systematic review (32 studies)

Study (year)	*N* (% male) ^*^	Mean age ± SD/age range	Health conditions	# of sample collections across 24 hours	Components of CBC included
Abo et al. (1978) [21]	6	23–45	Healthy	7	Lymphocytes
Ackermann et al. (2012) [22]	15 (100%)	23.7 ± 5.4/19–35	Healthy	16	Leukocytes, lymphocytes, monocytes
Akbane (1969) [23]	9 (55.5%)	9–43	Healthy	8	Eosinophils
Akbulut et al. (2003) [24]	10 (0%)	34^A^/20–48	Healthy	6	Leukocytes, neutrophils
Aoto et al. (2014) [25]	10 (100%)	30 ± 2.6/26–34	Healthy	4	Erythrocytes, leukocytes, platelets
Baldwa et al. (1977) [26]	10	16–25	Healthy	4	Basophils
Bates et al. (1994) [27]	18 (60%)	19–25	Asthma	4	Eosinophils
Dahl (1977) [28]	8	24–58	Healthy	8	Eosinophils
Dahl et al. (1978) [29]	9	21–66	Healthy	7	Eosinophils
Fournier et al. (2018) [30]	25 (56%)	24.3 ± 2.6/≥18	Healthy	6	Platelets
Grattan et al. (2003) [31]	10 (40%)	39^A^/24–63	Healthy	4	Basophils
Gresele et al. (1993) [32]	10 (40%)	22–34	Healthy	3	Platelets
Haus et al. (1990) [33]	10 (50%)	31 ± 11/21–50	Healthy	6	Eosinophils, lymphocytes, neutrophils
Jennings et al. (1990) [34]	12 (100%)	27.6/23–35	Healthy	7	Eosinophils, lymphocytes, neutrophils
Kanabrocki et al. (1991) [35]	2 (50%)	47	Healthy	11	Basophils, eosinophils, erythrocytes, leukocytes, lymphocytes, monocytes neutrophils, platelets
Kanabrocki et al. (1999) [36]	11 (100%)	55.2 ± 9.7/46–72	Healthy	8	Platelets
Kanabrocki et al. (1990) [37]	9 (100%)	44.3 ± 1.9/41–47	Healthy	8	Basophils, eosinophils, erythrocytes, hematocrit, hemoglobin, leukocytes, lymphocytes, monocytes, neutrophils, platelets
Krzyzanski et al. (2021) [38]	24 (0%)	22–37	Healthy	10	Basophils, neutrophils
Lasselin et al. (2015) [39]	9 (100%)	22–27	Healthy	14	Eosinophils, leukocytes, lymphocytes, monocytes, neutrophils
Manfredini et al. (1994) [40]	10 (50%)	65/24–84	Healthy	4	Basophils, eosinophils, erythrocytes, hemoglobin, leukocytes, lymphocytes, monocytes, neutrophils, platelets
Melchart et al. (1992) [41]	13 (100%)	23.7 ± 2.9^B^/21–32	Healthy	6	Eosinophils, erythrocytes, hematocrit, hemoglobin, leukocytes, lymphocytes, monocytes, neutrophils, platelets
Pavlishchuk et al. (1979) [42]	10		Healthy	6	Basophils, platelets
Postma et al. (1985) [43]	16 (100%)	53 ± 4.7/43–60	Chronic, partially reversible airflow obstruction	7	Leukocytes, lymphocytes
Reinberg et al. (1977) [44]	9 (66.7%)	23.8 ± 4.9/19–28	Healthy	6	Leukocytes
Rosenfeld et al. (1994) [45]	12 (100%)	18–22	Healthy	4	Hematocrit, leukocytes, platelets
Spector et al. (2012) [46]	12		Asthma	15	Eosinophils
Swoyer et al. (1989) [2]	150 (52.7%)	24 ± 10/14–34	Healthy	6	Basophils, eosinophils, erythrocytes, hematocrit, hemoglobin, leukocytes, lymphocytes, monocytes, neutrophils, platelets
Tornatore et al. (1998) [47]	5 (100%)	31 ± 5.8/24–37	Healthy	6	Lymphocytes
Touitou et al. (1986) [48]	7 (100%)	24 ± 3.9/19–31	Healthy	6	Erythrocytes, hematocrit, hemoglobin
Wempe et al. (1992) [49]	9 (67%)	24 ± 6.8/18–39	Episodic wheezing consistent with asthma	6	Eosinophils
Winkel et al. (1981) [50]	21 (47.6%)	37.4 ± 11.4/24–55	Healthy	6	Basophils, eosinophils, leukocytes, lymphocytes, neutrophils, monocytes
Zaslavskaia et. al (1974) [51]	50	23–72	Healthy	5	Platelets

^A^Median,

^B^SEM,

^*^if sex of participants not mentioned, then left blank in table.

With regards to study design, the studies controlled various factors known to affect circadian rhythms including inpatient or outpatient setting, activity levels, sleep, lighting, meal frequency, and blood draw conditions. Blood draws were not taken between midnight and 6 am in some studies of leukocytes (63.6%), erythrocytes (66.7%), platelets (63.6%), neutrophils (70%), lymphocytes (75%), monocytes (83.3%), eosinophils (85.7%), and basophils (28.6%). Fourteen studies were performed in the in-patient setting [2, 22, 25, 27, 30, 32, 36, 37, 39, 41, 43, 45, 47, 49], five were performed in the outpatient setting [21, 26, 35, 46, 50], one included patients from both settings [50], one was in the laboratory setting [44], one included only hospital employees [31], and 10 did not specify [23, 24, 28, 29, 34, 38, 40, 42, 48, 51]. Twelve studies allowed ambulatory movement [2, 21, 22, 24, 26, 30, 33, 35, 36, 39, 41, 50], two studies required patients to be sedentary [37, 45], and 18 did not specify [23, 25, 27–29, 31, 32, 34, 38, 40, 42–44, 46–49, 51]. Subjects were diurnally active in 13 studies [2, 22, 23, 27–30, 33, 36, 37, 39, 44, 48] while nineteen studies did not specify [21, 24–26, 31, 32, 34, 35, 38, 40–43, 45–47, 49–51]. Nine studies specified the subject rooms were dark at night for sleep [22, 29, 30, 36, 37, 41, 43, 48] and two studies specified subjects had daylight exposure [28, 29], and 23 did not specify [2, 21, 23–27, 31–35, 38–40, 42, 44–47, 49–51]. Subjects had three meals a day in 14 studies [2, 21–23, 30, 33, 35–38, 43, 44, 48, 50], two meals a day in one study [45], and 17 studies did not specify [24–29, 31, 32, 34, 39–42, 46, 47, 49, 51]. Seventeen studies used an indwelling-catheter to draw blood [21, 24, 25, 27–32, 34, 39, 41, 44, 45, 47, 48, 50], one utilized an intra-arterial cannula [43], and 14 studies did not specify [2, 22, 23, 26, 33, 35–38, 40, 42, 46, 49, 51].

Of the 32 studies included in the systematic review: 27 presented a peak value, 27 presented a trough value, and 31 presented corresponding timing for the components of the CBC measures. One study, Kanabrocki et al. (1991), was not included in the calculations of the average peak and trough times and values, as only mesor data was provided [36].

### Meta-analysis

Of the 32 studies, 19 included SD or SE and could be included in the meta-analysis with data depicted in the forest plots summarized in Table 2. Of those 19 studies, 12 included blood draws between midnight and 6 am. Supplementary Figure 8 summarizes the time points of blood draws in each of the included studies.

**Table 2.** Summary of peak and trough values from forest plot analysis, with peak time and trough times as weighted averages across studies

	Studies included to compute time (#)	Peak time	Trough time	Circular time *r*-value (peak, trough)	Studies included to compute peak and trough values (#)	Peak value (cells/µL)	95% CI	Trough value (cells/µL)	95% CI
**Leukocytes**	12	20:01	08:10	0.854, 0.772	6	7.08 × 10^3^	[6.09 to 8.08]	5.94 × 10^3^	[4.93 to 6.96]
**Erythrocytes**	6	10:26	17:45	0.907, 0.288	3	4.75 × 10^6^	[3.65 to 5.85]	4.47 × 10^6^	[3.47 to 5.47]
**Platelets**	11	18:55	05:57	0.932, 0.509	7	258.88	[233.15 to 284.62]	205.02	[164.09 to 245.95]
**Neutrophils**	10	19:46	06:19	0.941, 0.839	3	5268.11	[4590.25 to 5945.98]	4040.66	[3496.00 to 4585.32]
**Lymphocytes**	12	23:54	10:47	0.916, 0.856	7	2264.87	[1783.44 to 2746.31]	1598.91	[1230.12 to 1967.71]
**Monocytes**	6	20:47	08:37	0.777, 0.906	2	490.14	[427.27 to 553.01]	417.31	[354.52 to 480.11]
**Eosinophils**	14	03:23	12:13	0.807, 0.802	6	288.25	[168.15 to 408.35]	207.30	[121.93 to 292.68]
**Basophils**	7	17:42	06:34	0.735, 0.663	3	47.88	[39.97to 55.79]	31.48	[17.06to 45.91]

**Figure 2.**
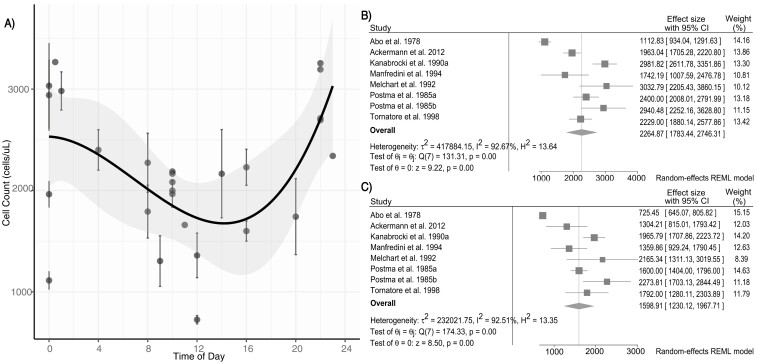
Rhythm of lymphocytes. (A) Lymphocyte values fitted with a third-degree polynomial generated via R statistical analysis. Standard error of the fit is shaded in gray around the line of best fit. For studies that reported standard error data, error bars are plotted with the associated data point (*p* = .010, *R*^2^ = 0.383, *n* = 11). The minimum point is at approximately 14:00 with a cell count of about 1700 cells/µL and the maximum point is at approximately 24:00 with a cell count of 2500 cells/µL (B) Forest plot of lymphocytes peak values created with Stata SE software. Six studies were not included in the forest plot of peak values due to lack of error data [2, 22, 33, 34, 39, 50]. (C) Forest plot of lymphocyte trough values created with Stata SE software. Six studies were not included in the forest plot of trough values due to lack of error data [2, 22, 33, 34, 39, 50].

The average peak of leukocytes is 7.08 thous cells/µL (95% CI: 6.09 to 8.08) at 20:01 (*r* = 0.854), with the average trough of 5.94 thous cells/µL (95% CI: 4.93 to 6.96) at 08:10 (*r* = 0.772). Of leukocyte subsets, notably the average peak of lymphocytes is 2264.87 cells/µL (95% CI: 1783.44 to 2746.31) at 23:48 (*r* = 0.916), with a trough of 1606.41 cells/µL (95% CI: 1174.63 to 2038.19) at 10:28 (*r* = 0.856). Lymphocyte peak/trough values from 12 studies in which they were available are plotted in Figure 2A along with a significant fitting third-degree polynomial function (*p* = .010), depicting rhythmicity of lymphocyte numbers. From seven studies, we constructed the forest plots depicted in Figure 2B and C.

**Figure 3.**
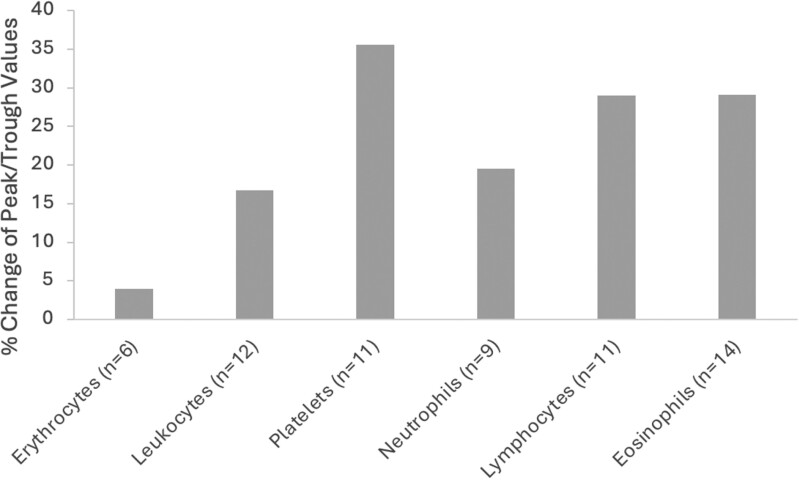
Bar graph showing the percent change in peak and trough differences for various components of a complete blood count (CBC) across 30 studies. The percent change is calculated as the difference between peak and trough values, divided by the peak value. The components include erythrocytes (3.94% change), leukocytes (16.77% change), platelets (35.56% change), neutrophils (19.48% change), lymphocytes (28.96% change), and eosinophils (29.13% change). Platelets show the highest percent change, followed by eosinophils and lymphocytes. Erythrocytes exhibit the smallest percent change.

Figure 3 shows a comparison of the weighted average peak and trough values from the 30 studies that included both peak and trough values. The values for basophils and monocytes are inconclusive given the limited data and are not included.

Of the 32 studies, 30 included peak/trough values and times for at least one component of the CBC and were included in the polynomial function analysis. Polynomial function analysis was performed for each component of the CBC and only lymphocytes, *p* = .010 (*n* = 11), erythrocytes *p* = .024 (*n* = 6), and platelets *p* = .043 (*n* = 11) showed statistically significant rhythmicity. Supplementary Table 2 summarizes linear or polynomial function estimates for each CBC component as well as the estimated maximum value, maximum time, and *P*-value. Supplementary Figures 1–7 graphically depict peak/trough averages from available studies, the linear or polynomial fit, and the forest plots aggregated from the studies for each component of the CBC.

## Discussion

In this systematic review and meta-analysis, we identified in 32 studies that most components of the CBC exhibit time of day differences, varying between 4% and 40% from peak-to-trough values across cell types in the studies we analyzed. Using a polynomial function analysis, we found the strongest 24-hour rhythm in lymphocytes and erythrocytes. This is consistent with previous literature of the mechanistic underpinning of cortisol rhythms driving lymphocyte trafficking. Whereby morning cortisol peak increases CXCR4 expression on lymphocytes thereby homing lymphocytes back into the bone marrow and decreasing peripheral counts [52, 53]. Consistent with this and previous publications, we found the time of peak peripheral lymphocyte count from a weighted average of 12 studies was a clock time of 23:54 and the trough at 10:47, with a change in value from peak to trough of ~29% [7, 52, 53].

In general, identified peak and trough times of the CBC components were in agreement with previously reported values [9, 10]. Based on the weighted averages of peak and trough times, erythrocytes, hemoglobin, and hematocrit peaked in the mornings and had troughs at night between 9 and 11 pm. Leukocytes, platelets, and neutrophils peaked in the afternoons between 6 and 9 pm and had troughs in the early mornings between 6 and 8 am with neutrophils and platelets exhibiting earlier troughs than leukocytes. Lymphocytes peaked around midnight and had a trough at 11 am, while eosinophils peaked even later, around 3 am, and had a trough at noon. Specifically, lymphocytes, erythrocytes, platelets, and neutrophils had the strongest association with their respective calculated peak times as each exhibited a *r* > 0.9. Given the limited data, the peak and trough times of monocytes and basophils are inconclusive.

We also noted heterogeneity in the peak and trough times of interrelated CBC components. The erythrocytes, hemoglobin, and hematocrit all peaked in the morning and had troughs in the evening, while cells of the myeloid lineage (neutrophils, eosinophils, and basophils) all peaked at various times in the evening. Interestingly, although platelets are of a different lineage, they had rhythmicity more aligned to the myeloid lineage than erythrocytes.

Our study also defined methodology for pooling studies or data sets to analyze rhythmicity of CBCs. Specifically, the forest plot analyses allowed comparison of peak and trough across the included studies which included standard deviation/standard error. This is complimented by a polynomial function analysis with a best-fit line testing a goodness of fit to a 24-hour period and providing more precise timing estimates (not limited to when data in the individual studies were collected). The polynomial function’s broad characterization of the rhythmicity of each CBC component permits visual comparison of the cell types and identification of the cell components with the most and least substantial rhythmicity. The choice of analysis for rhythmicity with a polynomial function was chosen as it is an objective, flexible, and reproducible method that can capture complex nonlinear data patterns. However, limitations of the polynomial function best-fit curve include poor accuracy when there is limited data between time points and the assumption of one maximum or minimum point for a second-degree polynomial and one maximum and one minimum point for a third-degree polynomial. Of note, erythrocytes and leukocytes had statistically significant rhythms based on the third-degree polynomial function analysis. Neutrophils were also best represented by a third-degree polynomial, but this function was not statistically significant. Leukocytes and platelets were best represented by a second-degree polynomial that was a statistically significant rhythm for platelets while leukocytes were approaching significance. Yet, interestingly platelets and eosinophils had among the highest percent change in peak to trough values which suggests a need for further analysis. Since eosinophils and basophils were well represented by both a second-degree polynomial and linear function, a linear best-fit line was chosen for ease of interpretation. Meanwhile, given the limited data available, monocytes were poorly fit with each model indicating that thus far multiple studies have not captured rhythmicity of monocytes. The linear function model for monocytes was included in the Supplementary Figures to demonstrate this inconclusive data and further highlight the need for more sense sampling in this CBC component population.

In terms of the estimated maximum and minimum times from the polynomial function analysis for lymphocytes, erythrocytes, and neutrophils, all relatively correspond to the peak and trough times calculated with circular statistics. The largest difference between the polynomial function estimate and the circular time calculation is 3.5 hours between the estimated minimum time for lymphocytes, although there is a lack of data between 2 pm and 6 pm which may be influencing these results. The estimated maximum based on the second-degree polynomial for leukocytes also closely reflects the calculated peak time.

Limitations to this review are significant and highlight the gaps in knowledge that still need to be filled. One limitation of the analysis of the peak and trough times was the number of studies that did not measure blood values after 12 am for nighttime values, which skews results even in a polynomial function analysis. Another limitation is the choice of including papers with three or more time points to balance data availability in the literature with methodological rigor. While this approach broadened the scope of the review, it potentially limited our precision in characterizing circadian rhythms. Although studies with three time points can detect rhythmic patterns through sinusoidal or polynomial curve fitting, they yield less reliable estimates of circadian parameters such as amplitude and phase. To address this limitation, we analyzed results minimal sampling cautiously and integrated them with more robust studies to develop a plausible meta-profile of CBC rhythmicity.

Despite the widespread use of the CBC and the established knowledge of the effect of diurnal rhythmicity on its values, our systematic review only identified 32 studies with sufficient data to compare the behavior of the CBC cell types in adults, for a total of 548 subjects. In our analysis, few CBC components had statistically significant results in either the polynomial function analysis or the forest plot due to the small sample size, the several studies without time points between midnight and 6 am, and the overall heterogeneity of the studies. Even given these challenges, the peak and trough times of the lymphocyte component of the CBC calculated in this study generally correspond to the expected rhythmicity [7, 9, 10].

Given the complexity of conducting controlled experiments with multiple blood draws over 24 hours, it is not surprising that 80% of the included studies had fewer than 20 total participants. Outcomes were not also reported in a standardized manner across studies, i.e. tables of cosinor data, graphs of cell count fluctuation versus individual data for each patient. Although the weighted average of the peak and trough values across all the studies for CBC component remained similar to the forest plot analysis results, one of the limitations of this systematic review is the significant number of studies that could not be included in the polynomial function or forest plot analysis due to lack of standard error or standard deviation reported in the studies. The weighted averages also did not account for the number of time points included in each study, thus studies with fewer time points and more participants could skew the data.

In summary, the current systematic review offers a detailed analysis of rhythmicity of the cell components of the CBC and their correlation to each other. We suggest that going forward trials for determining CBC reference values consider the time of day in their analyses. Further research is needed to ascertain the rhythmicity of the cell populations in larger study samples, particularly with samples taken throughout the day/night from the same participants. Further studies on the diurnal behavior of blood cell values are also needed across all age groups and within different diseased populations to gain a more complete understanding of the expected changes of each cell type in the age of precision medicine.

## Supplementary Material

zpae086_suppl_Supplementary_Figures_S1-S8_Table_S2

## Data Availability

No new data were generated or analyzed in support of this research.

## Prospero Protocol Registration

PROSPERO 2022: CRD42022308435.
