# Supplementary material for: Systematic review: differences in complete blood count component rhythms
Source: Sleep Adv. 2024 Dec 9;5(1):zpae086. doi: 10.1093/sleepadvances/zpae086 (PMC11672111; doi:10.1093/sleepadvances/zpae086)
Supplement: zpae086_suppl_Supplementary_Figures_S1-S8_Table_S2 [file zpae086_suppl_supplementary_figures_s1-s8_table_s2.pdf]

**Systematic Review: Differences in Complete Blood Count Component Rhythms**

Anna Busza[1], Vani Sharma[2], Kendall Ferguson[3], Andrea Fawcett[4], Justin Knoll[1], Marta Iwanaszko[5], Phyllis Zee[6], Anna Fishbein[7]

[1] *Northwestern University Feinberg School of Medicine, Chicago, Illinois, USA*

[2] *University of Illinois at Urbana Champaign, Champaign, Illinois, USA*

[3] *Northwestern University, Evanston, Illinois, USA*

[4] *Ann & Robert H. Lurie Children's Hospital, Chicago IL, USA*

[5] *Department of Biochemistry and Molecular Genetics, Northwestern University Feinberg School of Medicine, Chicago, Illinois, USA*

[6] *Department of Neurology and Center for Circadian and Sleep Medicine, Northwestern University Feinberg School of Medicine, Chicago, Illinois, USA*

[7] *Ann & Robert H. Lurie Children's Hospital Department of Allergy, Division of Pediatric Allergy & Immunology, Chicago IL, USA*

Anna Busza (anna.busza@northwestern.edu): 303 E Chicago Ave, Chicago, IL 60611

Vani Sharma (vshar5@illinois.edu): 901 W Illinois St, Urbana, IL 61801

Kendall Ferguson (kendallferguson2025@u.northwestern.edu): 833 Clark St, Evanston, IL 60208

Andrea Fawcett (afawcett@luriechildrens.org): 225 E Chicago Ave, Chicago, IL 60611

Dr. Justin Knoll (justin.knoll@northwestern.edu): 303 E Chicago Ave, Chicago, IL 60611

Dr. Marta Iwanaszko (m.iwanaszko@northwestern.edu): 303 E Superior St, Chicago, IL 60611

Dr. Phyllis Zee (pzee@nm.org): Abbott Hall, 5th floor, 710 N Lake Shore Dr., Chicago, IL 60611

Dr. Anna Fishbein (afishbein@gmail.com): 225 E Chicago Ave, Chicago, IL 60611

**Supplemental Table 2. Summary of fitted Peak Time and Values from Polynomial Function Analysis.** Polynomial function analysis was conducted using the ggplot2 package in the R statistical environment. Peak values were not calculated for cell types with second-degree polynomial fits that yielded a minimum value or with unreliable polynomial fits. Of note, monocytes were poorly fit with each model likely due to insufficient data. Monocytes remained included in the analysis to demonstrate the lack of rhythmicity captured in previous studies.

| | Studies Included (#) | Estimated Maximum Value (cells/uL) | Estimated Maximum Time | Best Fit polynomial ($1^{st}$ / $2^{nd}$ / $3^{rd}$ degree) | Best Fit p-value | Best Fit $R^2$ value | AIC / BIC |
|---|---|---|---|---|---|---|---|
| **Leukocytes** | 12 | 7313.31 | 20:27 | $2^{nd}$-degree | 0.067 | 0.273 | 361 / 365 |
| **Erythrocytes** | 6 | $5.31 * 10^6$ | 07:31 | $3^{rd}$-degree | 0.024 | 0.531 | 22.7 / 26.5 |
| **Hemoglobin** | 5 | -- | -- | $2^{nd}$-degree | 0.513 | -0.024 | 115 / 120 |
| **Hematocrit** | 5 | 42.47% | 11:05 | $2^{nd}$-degree | 0.172 | 0.122 | 159 / 165 |
| **Platelets** | 11 | -- | ~22:00 +/- 2 hours* | $2^{nd}$-degree | 0.043 | 0.310 | 219 / 223 |
| **Neutrophils** | 9 | 5160.98 | 17:21 | $3^{rd}$-degree | 0.135 | 0.286 | 336 / 341 |
| **Lymphocytes** | 11 | 2532.19 | 23:47 | $3^{rd}$-degree | 0.010 | 0.383 | 424 / 431 |
| **Monocytes** | 5 | -- | -- | $1^{st}$-degree | 0.916 | 0.0002 | 96.4 / 96.6 |
| **Eosinophils** | 13 | -- | -- | $1^{st}$-degree | 0.393 | 0.026 | 378 / 383 |
| **Basophils** | 6 | -- | -- | $1^{st}$-degree | 0.177 | 0.192 | 99.2 / 100 |

* Estimated time range of peak based on the fit. Resolution of +/- 2 hours based on available data points. Highest density of data points in this range (n =  4)

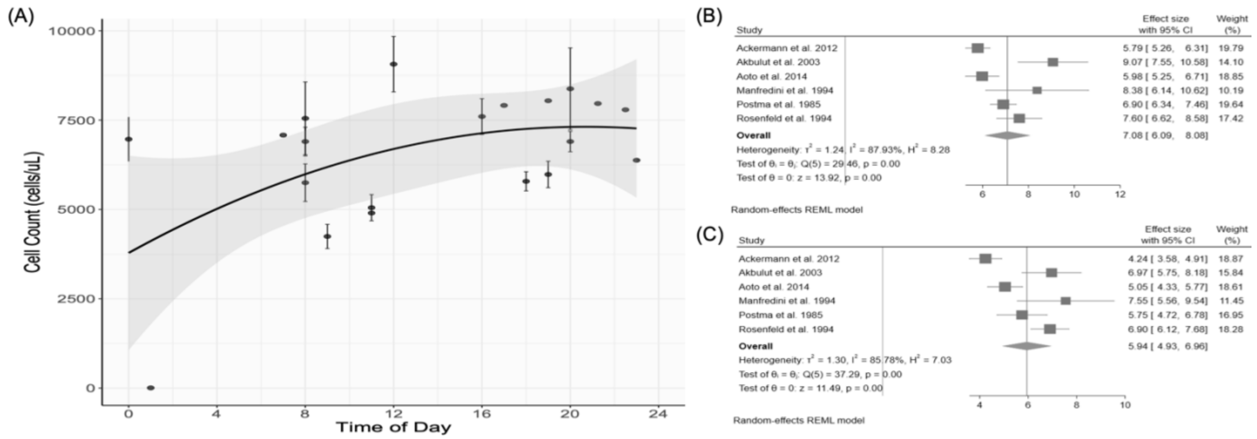


**Supplemental Figure 1. Rhythm of Leukocytes.** (A) Leukocytes values fitted with a second-degree polynomial generated via R statistical analysis. Standard error of the fit is shaded in grey around the line of best fit. For studies that reported standard error data, error bars are plotted with the associated data point (p=0.067, $R^2$=0.273, n=12) (B) Forest plot of leukocyte peak values created with Stata SE software. Seven studies were not included in the Forest plot of peak values due to lack of error data.[2, 23-25, 32, 34, 35] (C) Forest plot of leukocyte trough values created with Stata SE software. Seven studies were not included in the Forest plot of peak values due to lack of error data[2, 23-25, 32, 34, 35]

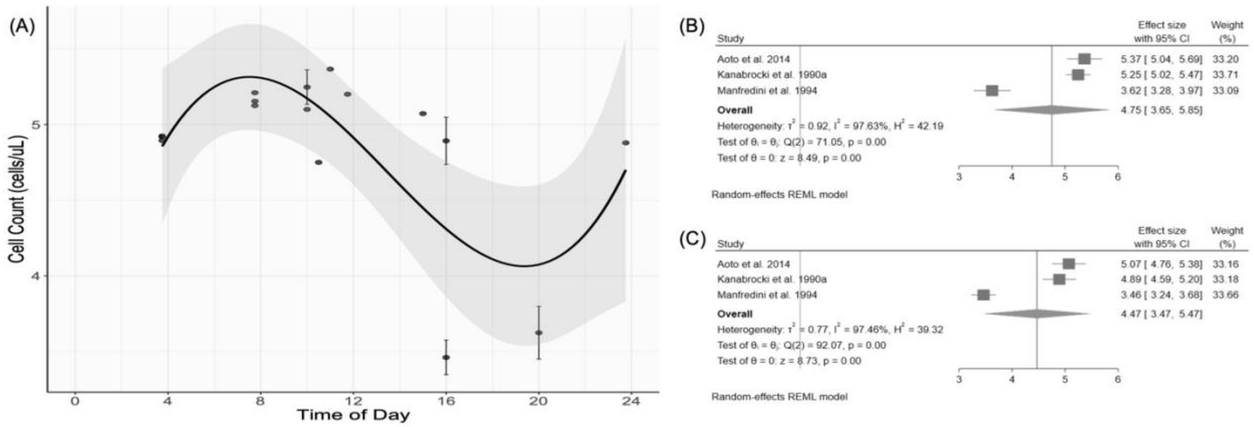


**Supplemental Figure 2. Rhythm of Erythrocytes.** (A) Erythrocyte values fitted with a third-degree polynomial generated via R statistical analysis. Standard error of the fit is shaded in grey around the line of best fit. For studies that reported standard error data, error bars are plotted with the associated data point. ($p=0.024$, $R^2=0.531$, $n=6$) (B) Forest plot of erythrocyte peak values created with Stata SE software. Four studies were not included in the Forest plot of peak values due to lack of error data.[2, 25, 32, 45] (C) Forest plot of erythrocyte trough values created with Stata SE software. Four studies were not included in the Forest plot of peak values due to lack of error data[2, 25, 32, 45]

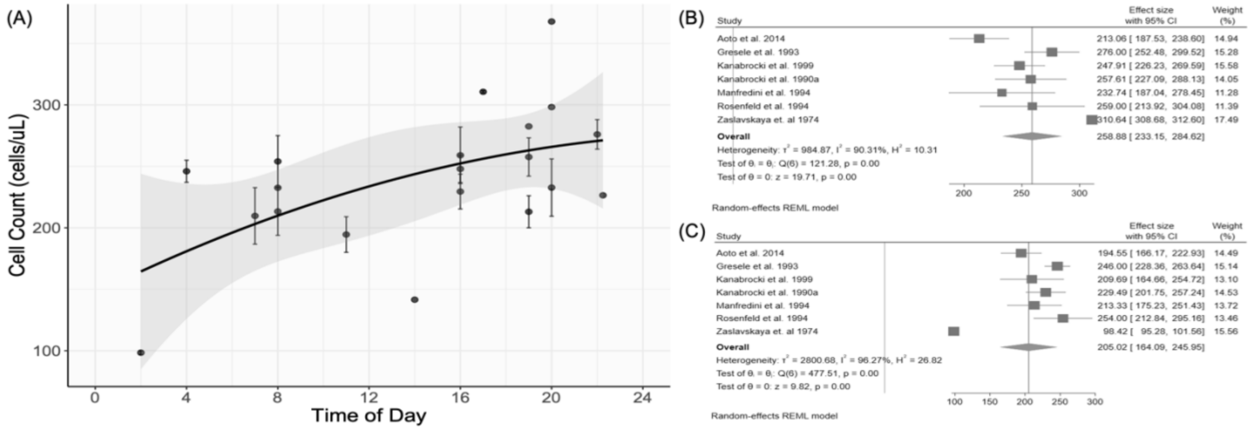


**Supplemental Figure 3. Rhythm of Platelets.** (A) Platelet values fitted with a second-degree polynomial generated via R statistical analysis. Standard error of the fit is shaded in grey around the line of best fit. For studies that reported standard error data, error bars are plotted with the associated data point. ($p=0.043$, $R^2=0.310$, $n=11$) (B) Forest plot of platelet peak values created with Stata SE software. Five studies were not included in the Forest plot of peak values due to lack of error data[2, 20, 25, 32, 44]. (C) Forest plot of platelets trough values created with Stata SE software. Five studies were not included in the Forest plot of peak values due to lack of error data[2, 20, 25, 32, 44]

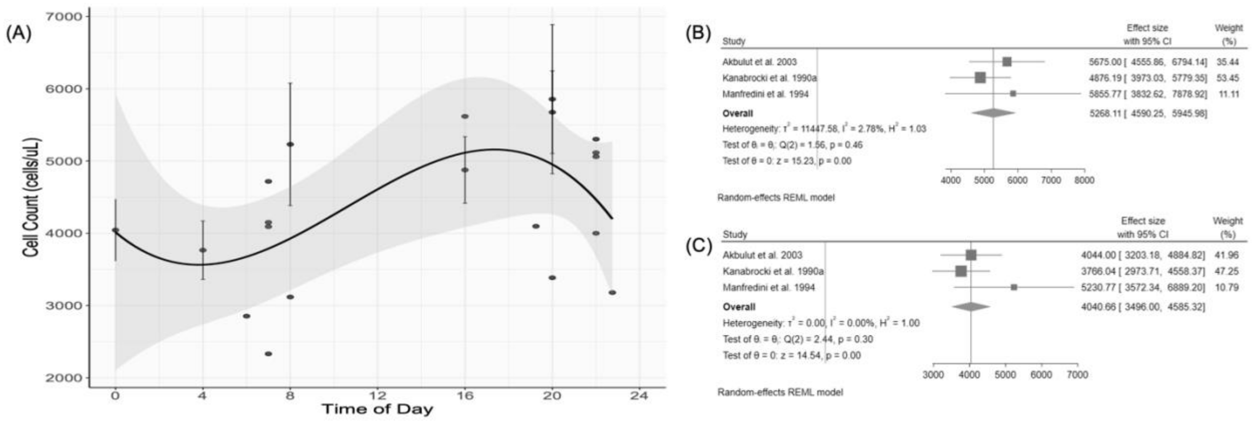


**Supplemental Figure 4. Rhythm of Neutrophils.** (A) Neutrophil values fitted with a third-degree polynomial generated via R statistical analysis. Standard error of the fit is shaded in grey around the line of best fit. For studies that reported standard error data, error bars are plotted with the associated data point. (p=0.135, $R^2$=0.286, n=9) (B) Forest plot of neutrophil peak values created with Stata SE software. Eight studies were not included in the Forest plot of peak values due to lack of error data.[2, 24, 25, 32, 34, 41, 42, 47] (C) Forest plot of neutrophil trough values created with Stata SE software. Eight studies were not included in the Forest plot of peak values due to lack of error data[2, 24, 25, 32, 34, 41, 42, 47]

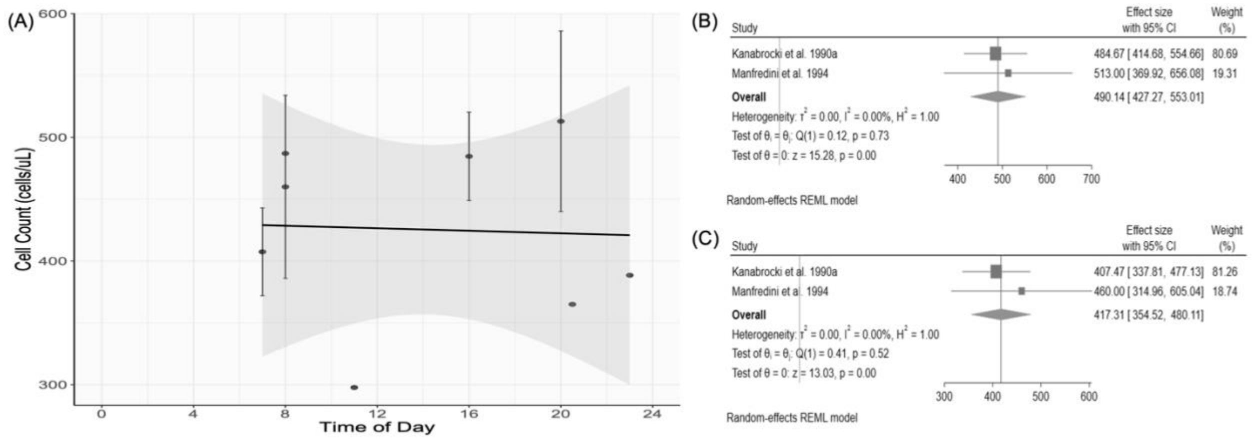


**Supplemental Figure 5. Rhythm of Monocytes.** (A) Monocytes values fitted with a linear function generated via R statistical analysis. Standard error of the fit is shaded in grey around the line of best fit. For studies that reported standard error data, error bars are plotted with the associated data point. (p=0.916, $R^2$=0.002, n=5) Of note, monocytes were poorly fit with 1st, 2nd, and 3rd degree models, likely due to insufficient data. Monocytes remained included in the analysis to demonstrate the lack of rhythmicity captured in previous studies. (B) Forest plot of monocyte peak values created with Stata SE software. Five studies were not included in the Forest plot of peak values due to lack of error data[2, 17, 24, 25, 32] (C) Forest plot of monocyte trough values created with Stata SE software. Five studies were not included in the Forest plot of peak values due to lack of error data[2, 17, 24, 25, 32]

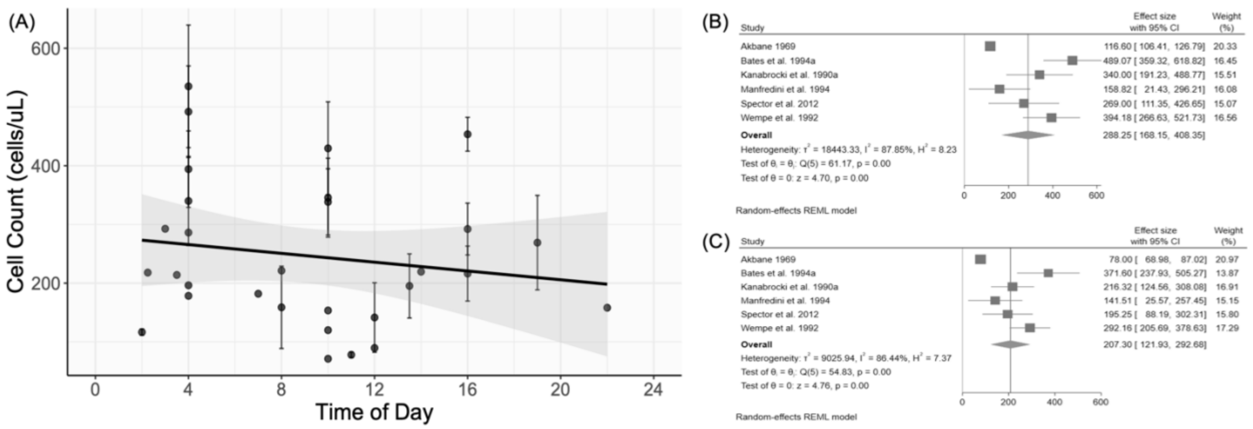


**Supplemental Figure 6. Rhythm of Eosinophils.** (A) Eosinophil values fitted with a linear function generated via R statistical analysis. Standard error of the fit is shaded in grey around the line of best fit. For studies that reported standard error data, error bars are plotted with the associated data point. ($p=0.393$, $R^2=0.026$, $n=13$) (B) Forest plot of eosinophil peak values created with Stata SE software. Nine studies were not included in the Forest plot of peak values due to lack of error data.[2, 24, 25, 32, 34, 39-41, 47] (C) Forest plot of eosinophil trough values created with Stata SE software. Nine studies were not included in the Forest plot of peak values due to lack of error data[2, 24, 25, 32, 34, 39-41, 47]

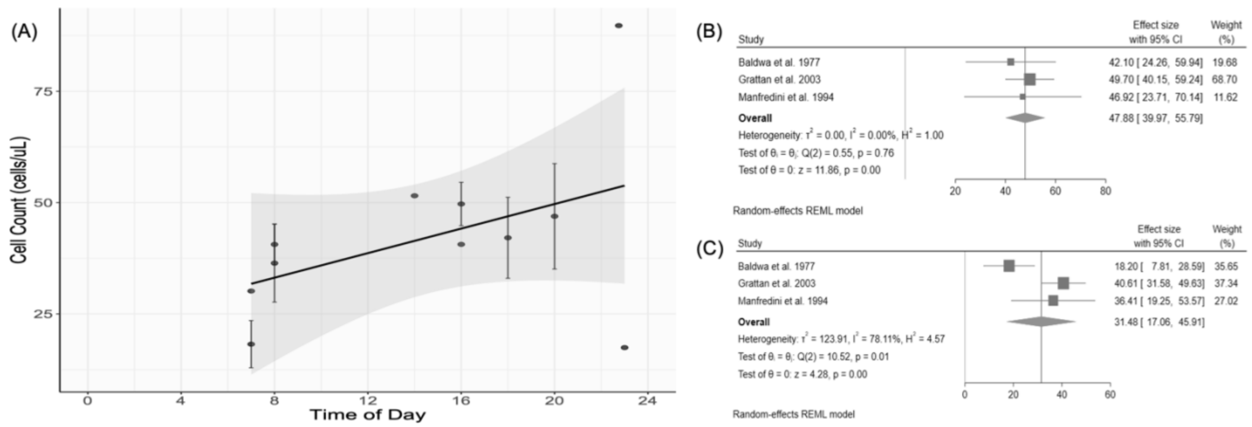


**Supplemental Figure 7. Rhythm of Basophils.** (A) Basophil values fitted with a linear function generated via R statistical analysis. Standard error of the fit is shaded in grey around the line of best fit. For studies that reported standard error data, error bars are plotted with the associated data point. (p=0.177, $R^2$=0.192, n=6) (B) Forest plot of basophil peak values created with Stata SE software. Five studies were not included in the Forest plot of peak values due to lack of error data.[2, 23, 32, 42, 44] (C) Forest plot of basophil trough values created with Stata SE software. Five studies were not included in the Forest plot of peak values due to lack of error data.[2, 23, 32, 42, 44]

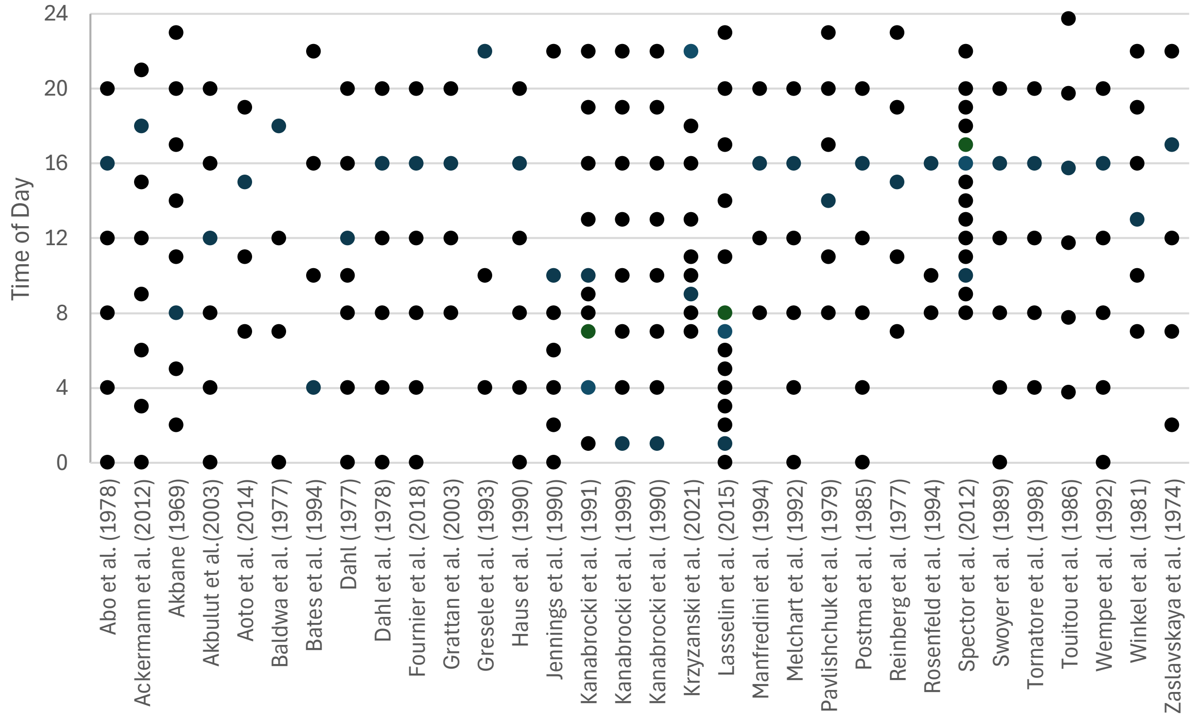


**Supplemental Figure 8. Time Points per Article.** The included time points for each article are depicted over a 24 hour period across the y-axis.
